# Arbuscular Mycorrhizal Fungi-Assisted Phytoremediation: A Promising Strategy for Cadmium-Contaminated Soils

**DOI:** 10.3390/plants13233289

**Published:** 2024-11-22

**Authors:** Shaopeng Zhao, Lei Yan, Muhammad Kamran, Shanshan Liu, Muhammad Riaz

**Affiliations:** 1 Guangdong Engineering and Technology Center for Environmental Pollution Prevention and Control in Agricultural Producing Areas, College of Resources and Environment, Zhongkai University of Agriculture and Engineering, Guangzhou 510225, China; zhaoshaopeng@zhku.edu.cn; 2 Institute of Biomedical Engineering, College of Life Sciences, Qingdao University, Qingdao 266071, China; yanlei2022@qdu.edu.cn; 3 School of Agriculture, Food and Wine, The University of Adelaide, Adelaide, SA 5005, Australia; muhammad.kamran@adelaide.edu.au; 4 College of Environment and Ecology, Hunan Agricultural University, Changsha 410128, China; suanlenyee@163.com

**Keywords:** Cd, AMF, heavy metal, tolerance, nutrient uptake

## Abstract

Arbuscular mycorrhizal fungi (AMF) have been shown to play a major role in regulating the accumulation, transport, and toxicity of cadmium (Cd) in plant tissues. This review aims to highlight the current understanding of the mechanisms by which AMF alleviate Cd toxicity in plants. Cd accumulation in agricultural soils has become an increasing global concern due to industrial activities and the use of phosphatic fertilizers. Cd toxicity disrupts various physiological processes in plants, adversely affecting growth, photosynthesis, oxidative stress responses, and secondary metabolism. AMF alleviate Cd stress in plants through multiple mechanisms, including reduced Cd transport into plant roots, improved plant nutritional status, modulation of organic acid and protein exudation, enhanced antioxidant capacity, and maintenance of ion homeostasis. AMF colonization also influences Cd speciation, bioavailability, and compartmentalization within plant tissues. The expression of metal transporter genes, as well as the synthesis of phytochelatins and metallothioneins, are modulated by AMF during Cd stress. However, the efficacy of AMF in mitigating Cd toxicity depends on several factors, such as soil properties, plant species, AMF taxa, and experimental duration. Further knowledge of the intricate plant–AMF–Cd interactions is crucial for optimizing AMF-assisted phytoremediation strategies and developing Cd-tolerant and high-yielding crop varieties for cultivation in contaminated soils.

## 1. Introduction

Heavy metal (HM) pollution has increased due to the growth of industry and human activities [1,2]. Certain metals, like Ni (nickel), Hg (mercury), Pb (lead), Zn (zinc), and Cd (cadmium), are categorized as HMs because they have a density higher than 5 g/cm^3^. HMs have contaminated millions of hectares of farmland in Europe [3]. Additionally, in China, more than 3.33 million hectares of soil are contaminated with HMs [4]. HMs in soils adversely affect agriculture and endanger public safety. Cd contamination of water, food, and air adversely affects human respiratory and digestive systems. “The International Agency for Research on Cancer” (IARC) classified it as a Group 1 human carcinogen in 1993 [5]. In Japan, rice with high levels of Cd has been linked to “Itai-Itai” disease [6]. Utilizing data from the 2004 soil geochemical survey and the 2014 soil monitoring data, researchers employed a Geographical Information System (GIS) model to predict the spatial and temporal variations of (*Cd_rice_*). The analysis revealed that, while the predicted (*Cd_rice_*) levels in 2014 remained below the food standard limit of 200 μg/kg, there was a significant increase observed over the decade. The median (*Cd_rice_*) content rose from 19.06 μg/kg in 2004 to 24.11 μg/kg (on a dry weight basis) in 2014 [7]. Due to the increasing soil Cd pollution, remediation measures are necessary to ensure the maintenance of sustainable agriculture and food security.

**Figure 1:**
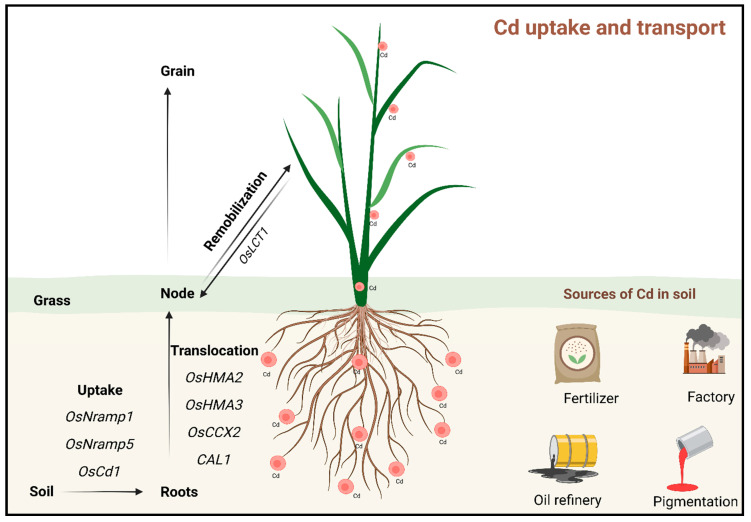
Cadmium uptake and translocation in plants. In plants, Cd typically accumulates in roots but can translocate to aerial parts like leaves, stems, and even seeds by different transporters, where it can cause toxic effects, such as oxidative stress, disruption of nutrient metabolism, and impaired growth. This process poses a significant risk for food safety and human health, as Cd can enter the food chain through contaminated crops.

Cadmium transference into plant tissues from soil can be detrimental, which can cause significant harm to various plant species [8]. Plant physiology involves a range of processes vital to plant health, such as reproductive functions, growth, metabolism, and overall vitality. Cd acts as a hazardous contaminant that negatively impacts these physiological processes [9]. Even at a low concentration, Cd is highly toxic to plants when administered in vivo, damaging cell membranes, disrupting mitochondrial functions, and inhibiting respiration, among other harmful effects [10]. When plants accumulate Cd concentrations of 5–10 mg per leaf of dry weight, Cd could induce lethal effects on the plants [10]. The growth of *Merwilla plumbea* plants was impacted by Cd stress, with a decrease in weight observed as Cd levels increased [11]. Similarly, *Radix isatidis* thrives under low Cd concentrations but faces growth suppression at higher levels [12]. Ref. [13] observed that Cd significantly reduces the biomass of *Phyllanthus amarus* and negatively impacts other plants by causing root discoloration and distortion. These changes inhibit growth, resulting in dwarf seedlings, damaged roots, and ultimately decreased yields. The harmful effects of Cd extend to several physiological functions, including osmoregulation, photosynthesis, and the antioxidant system [14,15,16,17]. Cd disrupts the photosynthetic apparatus, reduces photosynthetic rates, and inhibits pigment production [18]. Accumulation of Cd in vegetative organs hinders plant growth, leading to reductions in shoot and root length and overall weight [19]. Figure 1 shows how Cd accumulates in roots but can translocate to aerial parts like leaves, stems, and even seeds by different transporters.

The contamination by Cd presents a significant threat to agricultural productivity and food safety. To address this issue, researchers are exploring arbuscular mycorrhizal fungi (AMF)-based methods, which offer a potential solution for environmental clean-up [20]. AMF form a symbiotic association with plants, allowing them to obtain essential minerals from the soil in return for carbohydrates. AMF are classified as obligate biotrophs due to their dependence on higher plants for survival. AMF are essential for the development of plant roots [21]. Under uniform environmental conditions, the colonization rate of AMF decreases with increasing concentrations of Cd, with the greatest effect observed during the presymbiotic phase [22]. AMF improve plant nutrient and water absorption by trading carbohydrates, greatly affecting the balance and variety of ecological systems. The soil minerals are exchanged for carbon molecules (lipids and sugars) [23]. AMF promote plant growth and the ability to fight diseases and withstand environmental challenges like heavy metals, organic pollutants, and salt [24]. Through symbiotic partnerships, AMF may expand plant root surface area to uptake nutrients and water [25]. Understanding the mechanisms behind AMF’s role in mitigating heavy metal pollution can help reduce its ecological impact [26,27,28]. This knowledge is particularly crucial for ensuring food safety in regions affected by pollution from metalloids [29].

Using Tan’s [30] meta-analysis database, the authors expanded on the role of AMF in Cd bioremediation, highlighting their potential as effective bio-remediators. Tan’s [30] meta-analysis of 1,430 observations from 194 studies found that AMF inoculation reduced Cd and As content in both roots and shoots, especially during days 56–112. Legumes showed the highest reduction in metal uptake. AMF influence Cd absorption more than other factors by promoting extra-radical mycelia growth, which limits Cd uptake through root cell membranes and modifies rhizosphere pH, reducing Cd transport to host plants [30,31,32,33,34].

## 2. Role of AMF in Mitigating Heavy Metal Toxicity

One of AMF’s main mechanisms for eliminating Cd toxicity is immobilizing Cd in the soil matrix [19]. The mycelial network formed by AMF acts as a physical barrier that can sequester metal ions, making them less bioavailable to plant roots [35]. Additionally, the release of glomalin by AM fungi plays a crucial role in binding toxic metals, forming stable complexes that further reduce their uptake by roots [36]. Plants colonized by AM fungi exhibit increased activity of antioxidant enzymes, such as superoxide dismutase (SOD), catalase (CAT), and peroxidase (POD), under metal stress conditions. These enzymes effectively scavenge reactive oxygen species (ROS) generated during heavy metal-induced oxidative stress, thereby protecting plant cells from oxidative damage [24].

Mycorrhization stimulates the synthesis of phytochelatins, which are important for detoxifying heavy metals, within plant cells. By binding to metal ions, phytochelatins help mitigate the toxic effects of heavy metals and assist in their storage and transport within the plant [37]. These peptides have a high affinity for toxic metals and hence promote their detoxification in vacuoles. Furthermore, mycorrhizal symbiosis enhances the expression of root metal transporters, thereby influencing the uptake and translocation of heavy metals within plants. This increased expression can improve the plant’s ability to manage and tolerate metal stress [38]. AMF additionally promote plant nutrient uptake, specifically of phosphorus (P) and trace elements that can compete against heavy metals for available sites [39,40]. Therefore, by enhancing nutrition and strength in plants, AMF help relieve the harmful effects of heavy metals, including Cd toxicity, on plant growth and development [36]. Consequently, the symbiotic relationship between plants and AMF enhances soil structure and fertility, [41] thereby promoting healthy ecosystems under Cd stress [42].

## 3. Mechanisms of AMF-Induced Alleviation of Cd Toxicity

**Figure 2:**
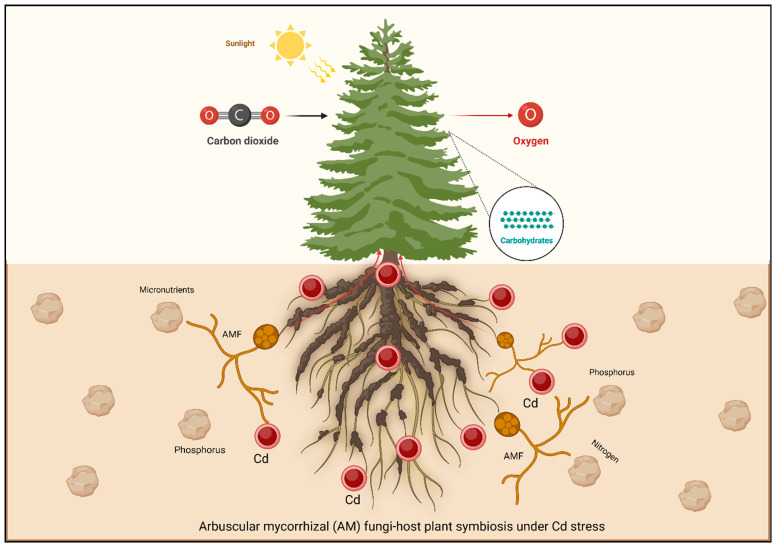
AMF hyphae sequester Cd in the rhizosphere through multiple approaches. “Arbuscular Mycorrhizal Fungi can help mitigate Cd accumulation in plant tissues through several mechanisms. By forming a symbiotic association with plant roots, AMF enhances the plant’s nutrient uptake like P, and improves its overall health, thereby reducing the need for excessive metal absorption. One key approach is through the immobilization of Cd in the soil: AMF hyphae sequester Cd in the rhizosphere, limiting its bioavailability and uptake by the plant. Additionally, AMF can induce changes in the root architecture and increase the expression of specific transporters, which selectively favor essential nutrients over toxic metals like Cd. AMF may also store Cd in fungal structures such as vesicles, preventing its translocation to aerial parts of the plant. Through these combined strategies, AMF plays a critical role in phytoremediation and promoting plant resilience to Cd stress”.

AMF can prevent the accumulation of Cd in plant tissues by various means. By synthesizing chelates, mycorrhizal plants could make stable metal–organic complexes that restrict Cd movement (Table 1). An illustration of AMF enhancing Cd stress tolerance in plants is presented in Figure 2.

### 3.1. AMF-Induced an Increase in Plant Growth

AMF symbiosis has the potential to improve the productivity of plants in Cd-polluted soil. For example, at concentrations of 50 mg/kg, Cd concentrations were decreased in various organs of *Populus yunnanensis*, leading to reduced Cd toxicity in *P. yunnanensis* [52], which increased cytokinin production and led to faster growth [53,54,55]. The root system of a plant, being the primary organ for absorbing HMs from the soil, is more sensitive to HMs than its aboveground structure. It was also observed that *R. irregularis* encouraged hemp development under Cd stress, leading to increased stem, leaf, and root biomass. This growth-promoting impact could be attributed to *R. irregularis* inoculation’s ability to mitigate plant damage caused by Cd stress [56]. Even as little as 25 mg/kg of soil Cd led to declines in biomass, yet hemp root biomass peaked at 80 mg/kg of soil Cd. Inoculation with AMF has been linked to enhanced photosynthetic efficiency under Cd stress. AMF can mitigate oxidative damage caused by heavy metals, which often leads to decreased chlorophyll content and impaired photosynthesis. By improving chlorophyll fluorescence parameters, AMF help maintain photosynthetic activity, which is crucial for growth and biomass accumulation [36,57,58,59].

### 3.2. AMF-Induced Changes in Soil Properties May Affect Cd Bioaccumulation

AMF contribute to soil structure by producing hyphae that bind soil particles together, forming stable aggregates. This process enhances soil aeration, water retention, and nutrient availability, which are crucial for plant health. The glomalin produced by AMF acts as a glue that stabilizes these aggregates, leading to improved soil conditions that can mitigate the leaching of contaminants like Cd during heavy rainfall events [60]. Some adsorption and binding mechanisms by mycorrhizal plants prevent Cd migration beyond the roots and mycorrhizal structures, thereby impairing Cd translocation and protecting shoots [21,38,61,62]. The soil pH exhibited a significant increase after AMF inoculation. The extraradical hyphae of AMF actively absorb anions “NO_3_^−^ and PO_4_^3−^” and release OH- ions as a consequence. Cd and anions “HPO_4_^−^, CO_3_^−^, OH^−^” result in the formation of compounds like Cd_3_(PO_4_)_2_, K_4_CdCl_6_, and CdCO_3_ [63]. Moreover, [64] reported associations between soil organic matter, anionic groups, and polycyclic aromatic hydrocarbons, resulting in lower Cd bioavailability. AMF increase the soil pH, causing negative charges on the solid phase, therefore reducing HM sorption onto oxide minerals. The increase in SOC content is attributed to greater inputs from root and hyphal activities [35,65]. The Cd retention into mycelium reduces Cd transfer to plants with the utilization of AMF–plant symbiotic systems [66].

AMF have been shown to improve soil water retention and hydraulic conductivity, allowing for better moisture availability to plants. This is especially important in conditions of variable rainfall, as it can mitigate the leaching of nutrients and heavy metals like Cd [67]. The release of substances that make metal unavailable for plant uptake through reduced pH and redox potential is a key role played by AMF in decreasing metal movement from contaminated soils into crops (soil organic matter content) [19,68,69]. Additionally, AMF-induced changes in soil properties may enhance the uptake and retention of HMs indirectly, which can limit their translocation from soil to plants [70]. AMF increase nutrient availability; for example, AMF immobilize Cd within soil via the growth of an extensive hyphal network that considerably binds Cd ions, making them inaccessible for root uptake. Root colonization by AMF enhances plant nutrition, especially phosphorus (P), which competes with Cd for root absorption sites, thus limiting its bioavailability [52].

Glomalin-related soil protein (GRSP) is a glycoprotein, synthesized by AMF, which plays a critical role in soil health and plant growth. This protein is significant for several reasons, particularly its ability to form complexes with HM and its impact on soil microorganisms [71,72]. Moreover, these complexes can improve plant-water relationships by stabilizing aggregates. This helps plants access water and nutrients from deeper layers within the soil profile via the AMF filament network [26,27,28]. Root exudates bind a great amount of HMs [39]. This will lead to a possible decrease in Cd of the rhizosphere, thus reducing Cd accumulation in mycorrhizal plant roots and subsequent Cd uptake. The specific surface area of extraradical mycelium of AMF can restrain hazardous toxic metals [73]. Furthermore, arbuscular, vesicle and intraradical mycelium can be utilized to sequester these toxic metals [74]. By enhancing the growth and nutrition of hosts, AMF lower the concentration of toxic metals in plant parts, producing a “dilution effect” [75,76]. Furthermore, some chemicals that are excreted by AMF hinder the uptake of metals by crops, hence modifying their availability through adsorption into the roots [77]. *G. versiforme*, *R. intraradices*, and *F. mosseae*, besides altering rhizosphere soil, also decreased Cu and Pb concentrations in corn [78]. By enhancing the growth and nutrition of hosts, AMF lower the concentration of toxic metals in plant parts [75,76].

AMF also control soil pH through the release of extracellular substances such as amino acids and glomalin, and they reduce soil Cd bioavailability [58,79]. AMF, for example, reduce soil Cd bioavailability by producing more glomalin in rhizospheres [37,49]. Moreover, *R. irregularis* can facilitate Cd uptake from the soil into cells of *Medicago truncatula* [80]. HM chelation can be facilitated by AMF due to their mycelial secretion constituents of cell walls and mycelium [81]. In a recent study, *G. mosseae* regulated Cd migration in the alfalfa rhizosphere soil [2]. In rhizosphere soils, substantial interaction took place between Cd and *G. mosseae*, as well as *Ph* and the *IRT1* gene, combined with the C/N ratio and the N value. Furthermore, alfalfa improved *G. mosseae* efficiency towards Cd clean-up [2].

### 3.3. AMF Enhanced the Accumulation of Nutrient Elements

AMF enhance the cycling of essential nutrients, particularly phosphorus, nitrogen, and micronutrients. They increase the bioavailability of these nutrients to plants, which can lead to improved plant growth and resilience against stress [60]. The production of glomalin, a protein secreted by AMF, contributes to the binding of soil particles, further enhancing nutrient retention. AMF also increased macro-elements like Mg, K, and Ca as well as trace elements such as Cu and Zn in *Zea mays* and *Medicago sativa*, respectively [31]. According to research, K and AMF supplementation could improve photosynthetic indices and plant chlorophyll production under Cd stress. Ca^2+^ and Cu^2+^ ions are among the essential nutrient elements that transiently compete with metals for binding sites during their transmembrane transport via metal transporter proteins [82,83].

Moreover, the increased nutritional elements compete with Cd in other metalloenzymes (for example, Cu^2+^ as a cofactor), which can be displaced by Cd. AMF indirectly induce plant tolerance by enhancing nutrients and water, modifying root structure, increasing shoot biomass, and alleviating oxidative stress [19,26,32,33]. Therefore, mycorrhizal symbiosis can develop defense mechanisms by maintaining ion homeostasis in response to abiotic stress [84]. Additionally, AMF increased Cu and Zn in poplar roots, while they did not affect macronutrients (K and Ca) in poplar shoots. This fluctuation in nutritional components between poplar shoots and roots, seemingly influenced by AMF, may contribute to ion homeostasis maintenance [31].

### 3.4. The Function of the AMF Hyphal Network

Moreover, the extraradical mycelium of AMF extends into the surrounding environment searching for water, minerals, or organic compounds. Fungal hyphae make a large area through which nutrients like nitrogen, phosphorus, and potassium can be taken up from the solution phase of the soil [85]. Fungal hyphae, through which nutrients are transported into plants, contribute to faster growth and enhanced nutrient uptake. These hyphae are enriched by mycorrhizal plants, which supply carbohydrates. Additionally, the mycelium of AMF increases nutrient supply within the plant and improves the stability of the soil structure. The hyphae produced by AMF act like a glue, binding soil particles together into stable aggregates. These aggregates form a porous medium that can be rapidly infiltrated by water and allow sufficient airflow for respiration [23]. When it comes to plants, this improved soil structure enables roots to penetrate more effectively, allowing for better aggregation. This is particularly beneficial for nutrient absorption, as it enhances the functionality of the root system, promoting its growth and development. By facilitating nutrient uptake, AMF contribute to the overall growth of the plant. Additionally, the mycelium from AMF helps repair soil clods, preventing surface crust formation that can lead to soil erosion and runoff. This contributes to better land management and erosion control [86].

AMF excrete exopolysaccharides or microbial extracellular polymeric substances (EPS), a mixture of simple carbohydrates, complex proteins, fatty acids, and other natural molecules, into the surrounding soil. These substances play several crucial roles in the soil–plant system, including nutrient retention, soil aggregation, water retention, and facilitating microbial interactions [87]. The activity of earthworm casts and plant roots further exposes organic residues, creating a physical interface with bacteria, which may lead to the decomposition of these residues [88]. EPS help create a soil matrix that enhances porosity, facilitating root growth, water infiltration, and nutrient exchange. This is because EPS absorb moisture like sponges in dry soil. Additionally, EPS play a key role in mediating interactions between AMF, plants, and microorganisms in the rhizosphere. Their complex structure creates a nutrient-rich environment for soil bacteria and fungi, thereby promoting microbial diversity and activity around plant roots [89].

Soil microorganisms can utilize EPS as a source of carbon and energy, promoting their growth and metabolic activity. Additionally, EPS are thought to function as signaling molecules that regulate microbial community dynamics and plant–microbe interactions within the rhizosphere. Furthermore, AMF-produced EPS play a role in sequestering heavy metals and pollutants in contaminated soils, reducing their bioavailability and toxicity to plants [90]. Heavy metals with positive charges can be bound by the negatively charged functional groups in EPS, forming stable complexes that are less mobile and less toxic to plants. This is one of the ways in which EPS contribute to the immobilization of heavy metals, aiding in the remediation of soils contaminated with these metals and mitigating their toxic effects on plants [31]. To improve soil management practices, boost crop productivity, and promote sustainable agriculture, it is essential to understand the dynamics and functions of the AMF hyphal network and EPS.

### 3.5. Oxidative Stress and Molecular Mechanisms

The non-enzyme and enzyme antioxidant systems respond differently across various plant species following AMF colonization. Under stress, there was a significant increase in POD, CAT, and GSH levels in the leaves (Gao et al. 2023) [42], while the roots exhibited an increase in POD activity. This enhancement of the antioxidant system helps reduce oxidative damage [91]. As a result, poplar leaves showed significantly lower levels of oxidative damage, H_2_O_2_, and malondialdehyde (MDA). The antioxidant system’s response to abiotic stresses varies depending on the treatment and the plant organ. For example, under 60 mg kg^−1^ Cd stress, mycorrhizal roots of *Lolium perenne* exhibited significantly higher SOD activity compared to the control, while their shoots showed a significant decrease in SOD activity [46].

AMF can enhance phosphorus and nitrogen uptake in alfalfa by collaborating with N-fixing bacteria and P-solubilizing microorganisms [92,93]. Cd, as an HMs ion, has the potential to disrupt the electrical balance of respiratory and photosynthetic chains through strong oxidation [61,94,95]. Cd can bind to proteins, causing denaturation and osmotic imbalances [96]. Plant metabolic processes rely on enzyme catalytic reactions and electron transfers located on cell membranes. The extent to which AMF can restore these rate-limiting nodes to alleviate Cd effects on plants remains an unanswered question.

The second study revealed that AMF regulate metal ion antagonism in plants and the function of divalent metal transport channels [97]. Various transporters are transcribed during this process, including Ca^2+^ channels, and K channels [98]. AMF can facilitate ion transfer and decrease Cd transference to aboveground portions. It has been reported that AMF decrease Zn^2+^ channels and promote Cu^2+^ antagonism to decrease Cd into leaf cells through Cu and Zn channels, as Cd can enter cells through these channels [99]. AMF can still promote Cd excretion from cells or into vacuoles, even if Cd is already present in them, by increasing the transcription of the *HMA3* genes and *HM-Pump11* of alfalfa.

Another study demonstrated that Cd exposure decreased citric acid and fumarate (including iso-citrate) in root cells, which may be related to its disruption of citrate synthase and succinate dehydrogenase [100]. High levels of succinic, oxalic, and malic acid may be due to the build-up of these organic acids as a result of a decrease in the tricarboxylic acid cycle following the Cd-mediated reduction of succinic acid, fumaric acid, and citrate. However, AMF stimulated the release of organic acids, which contributed to the production of bonded Cd in alfalfa [101,102]. Cd was more organically bound in AMF under Cd stress than in those exposed to Cd alone. Levels of PC, soluble protein content, and expression of the *HM-Res4* protein might increase as a result of AMF. The presence of these protein (or polypeptide) structures aids in reducing Cd by binding with it [103].

### 3.6. AMF Role in Cd Detoxification: Enhancing the Production of Phytochelatins

AMF play a crucial role in the induction of phytochelatins (PCs), which help reduce Cd toxicity and promote plant health, even in contaminated environments. One example of this is the synthesis of these metal-binding peptides by plants when exposed to heavy metals. These peptides are key components of the plant’s detoxification system [104]. PCs bind Cd ions to form stable complexes, which are then sequestered within vacuoles, thus protecting cells from the detrimental effects of Cd on cellular structures and processes [46].

It has been observed that AMF symbiosis enhances PC production and retention during Cd stress in plants. This can be explained by several factors related to the interactions between AMF and host plants. Firstly, phosphorus (P), an essential micronutrient involved in the biosynthesis of PCs, is enriched through AMF colonization. ATP, which contains phosphorus as a key component, provides the energy required for biochemical processes, including the synthesis of PCs [77]. Consequently, through the AMF association, improved P nutrition in plants leads to enhanced synthesis of PCs in response to Cd stress. Additionally, AMF symbiosis promotes the expression of genes associated with PC production in plant tissues. Some studies have shown that gene sequences regulating enzymes involved in the synthesis of PC precursors, such as γ-glutamylcysteine synthetase (*γ-ECS*) and phytochelatin synthase (*PCS*), are upregulated by AMF colonization [105]. These enzymes facilitate the formation of PCs by catalyzing the sequential addition of cysteine residues to glutathione molecules, ultimately forming polymeric chains. As a result, the upregulation of these genes in AMF-colonized plants leads to increased PC production, enhancing the plant’s ability to detoxify Cd and reducing Cd-induced toxicity [106]. In addition, the redox status within plant cells may be influenced by AMF symbiosis, which in turn affects PC biosynthesis and Cd detoxification. Excessive oxidative stress induced by Cd leads to the accumulation of ROS in plant cells, disrupting normal cellular processes and causing damage to biomolecules within the cells [35]. AMF induce the production of metallothioneins (MTs) and activate antioxidant enzymes, thereby enhancing plant resistance to HM pollution [76,107].

AMF hyphae can chelate Cd ions in the soil, making them inaccessible to plant roots. Additionally, AMF colonization can upregulate the transcription of Cd transporters in root cells, enhancing Cd sequestration and its accumulation in vacuoles, which reduces Cd translocation to shoots. This detoxification process through sequestration contributes to maintaining homeostasis within plant tissues [59]. It can be concluded that the enhanced Cd detoxification achieved through AMF symbiosis supports plant health in Cd-contaminated environments, highlighting the importance of AMF-mediated PC production for environmental sustainability and ecosystem resilience.

### 3.7. Coordination of Cd Uptake and Release Transporters by AMF Symbiosis Modulation

Different plasma membrane transporters in plants can facilitate the uptake of Cd by allowing Cd ions to enter root cells. Toxicity occurs when Cd accumulates over time within the plant tissues. In contrast, efflux transporters on the plasma membrane can help reduce Cd toxicity by pumping Cd ions out of root cells, preventing excessive accumulation and mitigating potential damage to plant tissues [32]. In addition, complex molecular mechanisms within the AMF–plant root symbiosis influence functional activities and protein synthesis related to Cd translocation. This symbiotic relationship can alter the expression levels of genes encoding Cd transporter proteins on root surfaces, resulting in modified patterns of Cd uptake and movement within plants [108].

The expression of Cd influx transporters in plant roots is reduced in the presence of AMF symbiosis. This restriction limits Cd ions from entering the cytoplasm or apoplastic space from the soil solution, effectively lowering internal Cd levels and, consequently, reducing toxicity to the plant [109]. In some cases, AMF symbiosis can also activate Cd efflux transporters that expel Cd ions from the roots into the surrounding soil. This efflux activity supports AMF-mediated Cd detoxification by preventing Cd accumulation in plant organs such as leaves and fruits, thus reducing potential toxicity and contamination [110].

**Figure 3:**
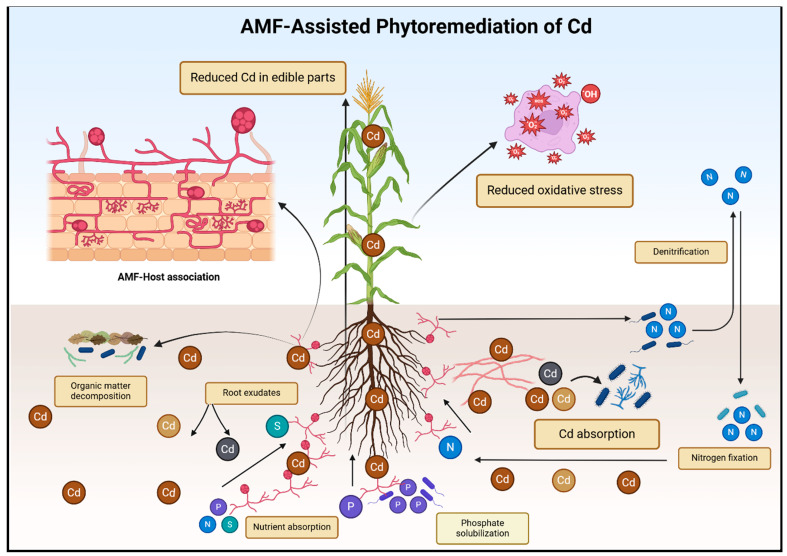
Illustration of AMF-assisted phytoremediation of Cd. Arbuscular Mycorrhizal Fungi (AMF) play a significant role in the phytoremediation of Cd and enhancing nutrient uptake while protecting plants. In contaminated soils, AMF-assisted phytoremediation works by sequestering Cd in the soil or fungal tissues, thus reducing its bioavailability and limiting Cd translocation to sensitive plant tissues. AMF also enhance the plant’s ability to cope with Cd-induced oxidative stress by boosting antioxidant enzyme activities and maintaining cellular integrity. This symbiotic relationship not only reduces Cd toxicity but also strengthens plant growth, improves root architecture, and fosters better overall resilience to environmental stresses. By combining enhanced nutrient uptake with heavy metal tolerance, AMF provide a dual benefit, aiding both in the detoxification of contaminated soils and in supporting healthier plant development.

Pathways modulating Cd transporter signaling through AMF symbiosis involve numerous complex molecular mechanisms that remain largely unknown. Exudates and metabolites from AM fungal hyphae can alter gene expression and transporter activity in host plants. These fungal signals may also interact with plant stress-response and nutrient homeostasis pathways, ultimately affecting Cd transporter expression and activity [111]. As a result, the cellular distribution and functionality of plant Cd transporters can be affected by AMF symbiosis. For instance, AMF may enhance the sequestration of Cd ions into vacuoles via increased activity of vacuolar transporters or through metal-rich vesicles. This compartmentalization reduces cytoplasmic Cd toxicity, thereby protecting cellular processes and aiding in detoxification [112]. Table 2 lists various AMF species that aid in heavy metal remediation through different mechanisms. Advanced knowledge is needed to optimize AMF-based strategies for Cd remediation in agricultural systems, with a focus on elucidating the specific molecular mechanisms and signaling pathways involved (Figure 3).

## 4. Factors Affecting AMF, Cd Uptake, and Plant Interaction

### 4.1. Factors Affecting Cd Persistence, Bioaccumulation, and Mobility

Cadmium, due to its persistence, bioaccumulation, high toxicity, extensive dispersion, and mobility in soils, poses serious threats to biodiversity, food security, and soil ecosystems. As a trace element, Cd remains prevalent in agroecosystems, where its impact is of particular concern [124]. Cd is taken up by plant roots and transported to other organs, leading to amplified and biomagnified effects on both humans and other living organisms [125,126]. Excessive levels of Cd can cause liver damage, anemia, renal failure, and nausea in humans [127]. Cd levels in soil and groundwater can be elevated. In addition to the total concentration, factors such as pH and redox potential significantly influence the mobility and availability of Cd in the soil solution. The main sources of Cd pollution in paddy soils include mining activities, the use of phosphatic fertilizers, and irrigation with untreated wastewater [128].

The presence and mobility of Cd are strongly influenced by factors such as soil pH, redox conditions, texture, and microbiological activity. The negative relationship between plant mobility and pH underscores the importance of soil pH in regulating Cd dispersion and bioavailability. Soil organic matter (SOM) forms complexes with Cd, thereby reducing its concentration and limiting its bioavailability [129]. Additionally, organic fertilizers can alter soil composition and porosity, influencing plant uptake and the cation exchange of Cd. One of the primary mechanisms by which PC chelates Cd is through the binding of cysteine sulfhydryl groups, forming Cd complexes that are then transported to the vacuole, reducing free Cd levels in the cytoplasm. Moreover, heterologous expression of the wheat gene *TaPCS1* has been shown to significantly enhance *Nicotiana glauca* tolerance to potentially toxic elements (PTEs), including Cd and Pb [57]. Unfortunately, overexpressing *AtPCS1* in *Nicotiana tabacum* increases human exposure to Cd through smoking, despite enhancing Cd accumulation and tolerance in the plant. Overexpression of the PCS gene makes plants more sensitive to Cd while simultaneously increasing their resistance to it. This highlights how various factors influence the modulation of the PC-mediated Cd stress response in plants [130]. The PC pathway, involving genes such as *PCS1* and *GSH1*, is crucial for how plants respond to Cd stress. Research suggests that HM transport genes also play a role in Cd uptake by plants. For example, *NRAMP1* in root internal cells facilitates the movement of Cd from root vascular cells to shoots, indicating that the *NRAMP1* response to environmental factors may influence Cd distribution and toxicity in plants [80]. In the rhizosphere, analyzing the influence of environmental parameters on Cd migration can reveal various approaches for phytoremediation.

### 4.2. Factors Affecting AMF Inoculation for Cd Translocation

Table 3 highlights several factors that can influence the effect of AMF on plant–mycorrhizal interactions. In long-term trials, the impact of AMF can be influenced by factors such as mycelial breakdown, decomposition, and the loss of soil resources [131]. Additionally, P fertilizers influence the effectiveness of AMF in reducing both shoot and root Cd accumulation. When soil P resources are abundant, host plants allocate fewer resources to their fungal partners, which can impair mycorrhizal function and mycelial development [132]. Therefore, the application of fertilizers containing AMF and P in heavy metal-contaminated soils could increase heavy metal uptake in crops. Consequently, using a combination of different types of AMF can better promote symbiotic associations with host plants, leading to improved mutualistic interactions that offer multiple functional benefits to the plants [133]. Additionally, combining different AMF species can influence the effectiveness of mycorrhizal symbiosis. Species occupying similar ecological niches may compete with each other, while those from diverse functional groups might exhibit cooperative benefits [134].

Previous studies have shown that AMF can reduce metal accumulation in plant tissues, although the effects vary across different species. These varying outcomes may result from differences in metal tolerance, as well as the physical, chemical, and structural characteristics of the AMF species involved [19]. Notably, the most effective species in reducing root Cd accumulation were *Rhizophagus irregularis* and *Diversispora versiformis*. Additionally, AMF activity in reducing Cd uptake may be influenced by the plant family. This could be due to a shared cellular program during symbiosis establishment, with legumes generally benefiting more from AMF associations than non-legumes [135,136]. Mycorrhizae can provide a better defense to plants compared to perennials. Additionally, annual plants with deeper root systems tend to exhibit a greater propensity for mycorrhizal colonization [137].

**Table 3:** Factors affecting AMF efficiency.

Levels	Factors	References
Abiotic factors at the community level	Soil properties	[138]
	Climate	[139]
	Light conditions	[140]
	Anthropogenic effects	[141]
Biotic factors at the community level	Plant community composition and competition	[142]
	AMF community composition	[143]
	Interaction with pathogen/parasites	[144]
Plant-derived factors at the organism level	Plant responsiveness and dependence on AMF regarding phosphorus uptake	[145]
	Carbon fixation capacity	[64]
AMF-derived factors at the organism level	Fungal nutrient mobilization capacity	[146]
	Nutrient transportation capacity	[147]
At the cellular/molecular level	Compatibility	[148]
	Signaling and gene expression	[149]
	Colonization ability and morphology	[150]
	Nutrient status like uptake and transport of phosphorus	[24]

## 5. Limitations and Challenges in Implementing AMF-Based Strategies for Cd Remediation

The implementation of strategies that use AMF for Cd mitigation in plants faces several limitations and challenges that must be addressed to enhance their effectiveness (Table 4). These challenges arise from various factors, including soil characteristics, Cd availability, plant-specific responses to Cd, as well as its mobility and translocation within plants. Additionally, issues such as the long-term stability of AMF inoculum in agricultural systems, practical application challenges, and regulatory and societal concerns need to be considered [151]. These limitations must be addressed to effectively apply AMF for Cd removal from soils. Soil properties play a critical role in determining the efficiency of AMF colonization and Cd remediation. The availability of Cd in the soil is another key factor influencing the success of AMF-mediated Cd removal. Other factors, such as soil pH, redox potential, and organic matter content, also affect the bioavailability of Cd. In soils with complex physicochemical properties or where Cd contamination is particularly high, it may not be feasible for plants or AMF to effectively immobilize or absorb Cd, reducing the potential for successful remediation [152]. Different plant species vary in their response to both AMF colonization and Cd stress. Some plants form strong mycorrhizal associations and accumulate more Cd than others. This information is crucial when selecting suitable plant species for phytoremediation of contaminated sites using AMF [153].

Challenges for AMF-based remediation include the dynamics of Cd and its mobility within the soil–plant continuum. Several processes, such as leaching, erosion, and plant uptake, contribute to the movement of Cd from one location to another. Relying solely on AMF to immobilize Cd may not be effective in preventing leaching or runoff from soils containing high levels of Cd, making it an inappropriate solution in some cases [154]. AMF may also affect the translocation of Cd within plants, leading to its redistribution among different tissues within individual plant organs. The long-term efficacy and stability of these strategies depend on whether sustained colonization and activity of these beneficial microorganisms can be maintained over time. However, land use changes, soil disturbances, and land management practices can disrupt AMF populations, potentially undermining efforts to remediate polluted areas [155]. 

However, practical considerations such as costs, scalability, and feasibility may limit the widespread adoption of AMF-based technologies for Cd cleanup in soils. Additionally, the high cost of inoculating AM fungi onto soil surfaces at large scales requires a significant labor force, further hindering the large-scale implementation of these techniques [156]. To overcome this limitation, it is recommended to promote native AMF rather than focusing on a single species or consortia applicable across diverse environments and crop species [157,158]. Regulatory requirements and public perception can also impact the acceptance and implementation of AMF-based Cd remediation strategies, emphasizing the need for stakeholder engagement. Ensuring safety and efficacy through risk assessment protocols and monitoring procedures is essential to comply with environmental regulations. Educating the public about these strategies is also crucial to foster active participation in the remediation process [159]. To overcome these limitations, interdisciplinary studies, collaborative efforts, and innovative approaches are essential.

**Table 4:** Various challenges for AMF and strategies to overcome them.

Challenges	References
Crop rotation with nonmycorrhizal crops can hamper AMF symbiosis, diversity, and activity	[160]
Fungicides or pesticides can have effects on AMF germination, colonization, and symbiosis	[161]
High nitrogen concentration in chemical fertilizer can inhibit AMF colonization	[162]
High phosphate concentration in chemical fertilizer can inhibit AMF symbiosis	[163]
Intensive tillage can affect AMF diversity and activity	[164]
*The Way Forward*	
Acquiring information on both the positive and negative effects of AMF technology on plants	[165]
Developing strategies to elevate the various parameters that influence the efficacy of AMF colonization in plants	[166]
Dissecting the molecular mechanisms of AMF–plant interactions by developing a genome-scale model that mimics the metabolic state of the plant with the integration of omics data	[167]
Employing gene editing tools (like CRISPR, and TALENs) to manipulate the gene of interest	[168]
Making use of advanced mathematical tools that can correlate the influence of AMF in casual pathways to understand the complexity of AMF–plant interactions	[169]

## 6. AMF Applications, Research, and Optimization for the Future

There are several unresolved issues and areas for improvement in AMF applications in plants. These range from fundamental AMF biology to applied research on agricultural practices and environmental sustainability [170]. Studying AMF diversity and functionality: Gaining a deeper understanding of AMF diversity, ecology, and genetics is essential for optimizing their use in plant–microbe interactions. Research efforts may focus on characterizing the genetic variability among different AMF species or strains, exploring their ecological roles in various soils, and uncovering the molecular mechanisms that govern their symbiotic interactions with plants [171].

Maximizing the efficiency and performance of AMF applications in crop systems will require the development of innovative methods and formulations for inoculation (Table 4). Additional studies should explore new delivery modes, such as seed coatings, root dips, or soil amendments, aimed at enhancing the establishment and persistence of AMF colonization in plant roots [172]. Enhancing crop resilience to environmental stresses through AMF-mediated stress tolerance has been a success story. Some studies have focused on how plants tolerate various abiotic stresses, such as drought, salinity, and heavy metal contamination, via AMF colonization, while others have sought to understand the molecular and physiological mechanisms underlying this stress mitigation [173]. Analyzing the interactive effects between AMF symbiosis and other stress management strategies may reveal synergistic or antagonistic relationships that influence plant health and productivity. However, logistical, economic, and practical challenges must be addressed before these laboratory successes can be scaled up to agricultural fields [174].

To interpret research findings into practical solutions and promote the global adoption of sustainable agronomy and environmental management practices, collaborative efforts must involve researchers, farmers, policymakers, and other industry stakeholders. In general, future studies on the use of AMF in plants hold significant potential for addressing critical issues in agriculture, environmental protection, and food safety. By deepening our understanding of AMF biology, optimizing inoculation methods to enhance plant–AMF interactions, and scaling up field applications, we can unlock their full potential to strengthen agro-ecosystems and better serve humanity.

## 7. Conclusions and Prospects

This review discusses the current understanding of the multifaceted mechanisms through which AMF help plants mitigate Cd stress and toxicity. The global extent of agricultural soil contamination by Cd has raised public health concerns regarding food safety and environmental pollution. AMF form symbiotic associations with the majority of land plants and play a pivotal role in enhancing the resistance of host plants to Cd and other heavy metal stresses.

AMF employ diverse strategies to reduce Cd accumulation in plant tissues, including binding Cd ions in fungal structures, modulating Cd speciation and bioavailability in the soil, competing with root Cd uptake, and reducing root-to-shoot translocation. Cd uptake and toxicity are reduced through AMF-induced improvements in plant nutritional status, as well as the maintenance of ionic balance and homeostasis. AMF trigger antioxidant defense mechanisms in plants, enhancing ROS scavenging capacity to counter Cd-induced oxidative damage. Additionally, AMF stimulate the exudation of organic acids and proteins, promoting Cd chelation and complexation. Furthermore, AMF colonization alters the expression of genes involved in phytochelatin synthesis, metallothionein production, and heavy metal transport in host plants.

However, substantial variations exist among different AMF taxa in their protective effects against Cd, depending on their tolerance mechanisms, hyphal networks, and colonization efficiencies. Overall, a multifaceted network of physical, biochemical, and molecular processes is modulated during the AMF–plant symbiosis to limit Cd accumulation and toxicity in plants growing in contaminated environments.

While significant progress has been made in unraveling AMF–plant–Cd interactions, several knowledge gaps persist that warrant further investigation. Detailed studies are needed to elucidate the signaling pathways involved in AMF-induced Cd resistance, including plant hormones and second messengers like calmodulin. The effects of AMF inoculation on metal transporter gene expression in various plant species and cultivars should be explored to identify potential genetic loci for breeding Cd-tolerant crop varieties. Optimizing AMF application protocols and formulations under field conditions is critical for the effective transfer of this eco-friendly biotechnology from controlled environments to agricultural soils.

Synergistic combinations of AMF inoculation with other sustainable approaches, such as bioremediation and organic amendments, should be evaluated at the field scale for cost-effective management of Cd-polluted soils. Further research is required to determine the long-term impacts of AMF inoculation on natural plant–microbe assemblages and soil ecological resilience in metal-contaminated environments. Advancing knowledge in these key areas will facilitate the transformation of AMF-assisted green technologies into practical solutions for enhanced food safety, agricultural sustainability, and ecosystem health.

## Figures and Tables

**Table 1:** Key mechanisms underlying AMF-mediated tolerance in plants under Cd stress.

Mechanistic Basis of AMF for Cd Stress Tolerance	References
Inhibited Na-transport; increased calcium/sodium ions ratio	[43]
Stimulated and altered root system morphology.	[44]
Enhanced hydraulic conductivity of the root at low water potential	[45]
Enhanced water absorption through the hyphal network	[46]
Improved uptake and metabolism of nutrients (like nitrogen and magnesium)	[44]
Increased contents of chlorophyll and carbonic amhydrase	[47]
Improved gas exchange capacity and stomatal conductance	[21]
Increased nitrate assimilation (through increasing nitrate uptake and nitrate reductase activity)	[21]
Improved antioxidant defense system	[38]
Increased the accumulation of organic acids (oxalic acid, fumaric acid, malic acid, and citric acid)	[48]
Increased pools of osmolytes/Osmo protectants (proline, glycine, betaine)	[49]
Improved cytokinin concentration and a higher translocation of photo synthetase	[50]
Increased performance of PS I and PS II	[51]

**Table 2:** Effect of AMF in the remediation of Cd.

Common Name	Scientific Name	Host Plant	Type of Fungi Concentration	Duration	Media	Main Effects	Reference
Arbuscular mycorrhizal fungi	*Glomus, Scutellospora*, and *Claroideoglomus*	*Robinia pseudoacacia* L.	0, 0.45, and 4.5 mg Cd kg dry weight soil^−1^	135 days	Soil	Increased antioxidant system	[113]
Common reed	*Phragmites australis*	*Funneliformis mosseae*	0, 5, and 20 mg Cd/L	60 days	Semi-hydroponics	AMF-stimulated Cd uptake via the iron pathway	[50]
Alfalfa	*Medicago sativa* L.	*Glomus mosseae*	0 and 8 mg Cd kg dry weight soil^−1^	6 months	Soil	Improved Cd bioconcentration and removal in the rhizosphere soil.	[2]
Maize	*Zea mays* L.	*Claroideoglomus etunicatum*	5 mg kg^−1^ Cd and 100 mg kg^−1^ La	60 days	Soil	Plants grew better	[114]
Hemp	*Cannabis sativa* L.	*Rhizophagus irregularis*	80 mg/kg	60 days	Soil	Increased plant development	[115]
Poplar	*Populus yunnanensis Dode*	*Funneliformis mosseae*	50 mg kg^−1^	60 days	Soil	The concentration of Cd in poplar plants was reduced	[52]
Alfalfa	*Medicago sativa*	*Glomus mosseae*	2 mg/kg	28 days	Soil	Decreased Cd absorption into shoots and protoplasm	[21]
Rice	*Oryza sativa* L.	None	0 and 5 mg Cd/kg soil	3 months	Soil	PLA at 2% caused a 28% decrease in shoot biomass, while PET had a less significant inhibitory effect.	[116]
Upland rice	*Oryza sativa* L.	*Glomus versiforme (Gv)*	10 μg Cd g^−1^ soil	10 weeks	Soil	Less Cd stored in roots and shoots	[108]
Kenaf	*Hibiscus cannabinus*	*Rhizophagus aggregatus*	0, 10, and 50 mg kg^−1^ Cd	60 days	Soil	More plant growth; less Cd build-up in roots and shoots	[44]
Alfalfa	*Medicago sativa* L.	*Glomus mosseae*	8.0 mg Cd kg dry weight soil^−1^	120 days	Soil	Improved plant growth	[42]
Maize	*Zea mays*	*Funneliformis mosseae*	20 mg·L^−1^	60 days	Sand column	68.1% increase in Cd uptake by maize	[117]
Salt-tolerant grasses	*Leymus chinensis, Puccinellia distans, Astragalus adsurgens*	*Funneliformis mosseae*	2 mg Cd per kg soil	12 weeks	Saline soil	The shoots showed a rise in biomass	[112]
Upland rice (Hanyou 3)	*Oryza sativa*	*Funneliformis mosseae, Rhizophagus intraradices*	33 mg/kg	50 days	Soil	Lower Cd concentrations in their shoots and roots	[82]
Upland rice (Hanyou 3)	*Oryza sativa*	*R. intraradices*	0.05 mM in water	105 days	Soil	Less Cd in their cell walls	[118]
Rice (Beidao 4)	*Oryza sativa*	*F. mosseae* *Rhizophagus irregularis*	0.5, 1, 2, and 5 mg/kg	90 days	Soil	Reduced sensitivity to Cd	[119]
Upland rice	*Oryza sativa*	*Glomus versiforme*	5 mg/kg	115 days	Soil	Lower Cd levels in upland rice	[120]
Rice (Zhenghan 9)	*Oryza sativa*	*F. mosseae* *R. intraradices*	0.05 mM and 0.1 mM in water	60 days	Soil	AMF can reduce Cd by rice plants.	[121]
Upland rice	*Oryza sativa*	*F. mosseae* *R. intraradices*	2 and 10 mg/kg	105 days	Soil	Reduced Cd in the cell wall and increasing Cd in vacuoles	[35]
Upland rice	*Oryza sativa*	*R. intraradices*	2 and 10 mg/kg	145 days	Soil	AMF can change the chemical forms of Cd in rice plants, such as by converting Cd into less toxic forms	[122]
Upland rice	*Oryza sativa*	*F. mosseae*	1.12 mg/kg	56 days 112 days	Soil	Reduced Cd in grains and aids in the phytoremediation of Cd-polluted soil	[123]

## Data Availability

No new data were created or analyzed in this study.
